# Virus-induced down-regulation of *GmERA1A* and *GmERA1B* genes enhances the stomatal response to abscisic acid and drought resistance in soybean

**DOI:** 10.1371/journal.pone.0175650

**Published:** 2017-04-18

**Authors:** Takuya Ogata, Yukari Nagatoshi, Noriko Yamagishi, Nobuyuki Yoshikawa, Yasunari Fujita

**Affiliations:** 1 Biological Resources and Post-Harvest Division, Japan International Research Center for Agricultural Sciences (JIRCAS), Tsukuba, Ibaraki, Japan; 2 Plant Pathology Laboratory, Faculty of Agriculture, Iwate University, Morioka, Iwate, Japan; 3 Graduate School of Life and Environmental Sciences, University of Tsukuba, Tsukuba, Ibaraki, Japan; Institute of Genetics and Developmental Biology Chinese Academy of Sciences, CHINA

## Abstract

Drought is a major threat to global soybean production. The limited transformation potential and polyploid nature of soybean have hindered functional analysis of soybean genes. Previous research has implicated farnesylation in the plant’s response to abscisic acid (ABA) and drought tolerance. We therefore used virus-induced gene silencing (VIGS) to evaluate farnesyltransferase genes, *GmERA1A* and *GmERA1B* (*Glycine max Enhanced Response to ABA1-A* and *-B*), as potential targets for increasing drought resistance in soybean. *Apple latent spherical virus* (ALSV)-mediated *GmERA1*-down-regulated soybean leaves displayed an enhanced stomatal response to ABA and reduced water loss and wilting under dehydration conditions, suggesting that GmERA1A and GmERA1B negatively regulate ABA signaling in soybean guard cells. The findings provide evidence that the ALSV-VIGS system, which bypasses the need to generate transgenic plants, is a useful tool for analyzing gene function using only a single down-regulated leaf. Thus, the ALSV-VIGS system could constitute part of a next-generation molecular breeding pipeline to accelerate drought resistance breeding in soybean.

## Introduction

Climate change has increased the frequency and severity of diverse abiotic stresses worldwide [1, 2]. Drought is by far the most devastating abiotic stress affecting plant growth and productivity [3]. In soybean (*Glycine max* L.), drought reduces seed quality and quantity, and induces changes in plant morphology, by reducing CO_2_ assimilation, leaf area development, and symbiotic N_2_ fixation [4,5]. Functional validation of candidate genes for improved drought resistance identified in model plants such as *Arabidopsis thaliana* could provide gene resources for breeding varieties of non-model staple crops, such as soybean, that can withstand drought conditions. Based on our knowledge of the mechanism underlying abscisic acid (ABA)/stress-signaling during the drought response in *Arabidopsis* [6–8], we have embarked on a study to generate transgenic soybean plants to validate the involvement of various candidate genes in stress signaling pathways [9–11]. As soybean, a partially diploidized tetraploid [12], is recalcitrant to transformation [13] and gene expression analysis, we employed virus-induced gene silencing (VIGS) to validate potential candidate genes for drought resistance in soybean.

VIGS is a post-transcriptional gene silencing-based technique for functionally characterizing plant genes through the knockdown of endogenous target gene expression [14–16]. Three plant viruses, *Bean pod mottle virus* (BPMV)[17], *Cucumber mosaic virus* (CMV)[18], and *Apple latent spherical virus* (ALSV)[19], have been used to perform VIGS in soybean. Soybean is not a natural host of ALSV, which was originally isolated from an apple tree in Japan [20]. ALSV has isometric particles that contain two ssRNA species, RNA1 and RNA2 [21, 22]. The ALSV-RNA2 VIGS vector can be engineered to harbor additional DNA fragments for VIGS analysis [23, 24].

The plant hormone ABA coordinates the plant’s responses to reduced water availability and influences diverse developmental processes [25–27]. Seed maturation and osmotic stresses such as drought and high salinity result in cellular dehydration, which increases endogenous ABA levels to trigger multiple developmental and physiological responses, including stomatal closure and changes in gene expression [6, 28]. Many key regulators of ABA signaling have been identified using forward and reverse genetic approaches, primarily in *Arabidopsis*. The *Arabidopsis* ABA-hypersensitive mutant *era1* (*enhanced response to ABA1*), harboring a disruption or deletion of the gene for the β-subunit of protein farnesyltransferase [29–32], exhibits enhanced ABA-induced stomatal closure due to the activation of a guard cell S-type anion channel and increased cytosolic Ca^2+^ levels in guard cells [29]. Although the role of ERA1-mediated protein farnesylation remains unclear, farnesylated CYP85A2 (a cytochrome P450 enzyme implicated in brassinosteroid biosynthesis) and ASG2 (a WD40 protein involved in seed germination) proteins have recently been shown to act as negative regulators of ABA responses in *Arabidopsis* [33, 34]. Furthermore, repression of *ERA1* has been shown to enhance drought resistance in *Arabidopsis* [31, 35], *Brassica napus* (canola) [35, 36], *Triticum aestivum* (wheat) [37], and *Oryza sativa* (rice) [38]. Therefore, *ERA1* represents a promising candidate gene for increasing drought resistance in soybean.

Here, we employed VIGS to evaluate whether *GmERA1A* and *GmERA1B*, which are homologs of *Arabidopsis ERA1*, enhance drought resistance in soybean. Soybean leaves subjected to ALSV-mediated *GmERA1s*-down-regulation showed an increased stomatal closure response to ABA and reduced water loss, gas exchange, and wilting under water-limiting conditions compared to control plants. These results suggest that *GmERA1*s act as negative regulators of ABA signaling during stomatal responses in soybean under dehydration conditions. These findings support the proposal that *ERA1* can be downregulated to increase drought resistance in soybean. In addition, they demonstrate that the ALSV-VIGS system is a useful tool for evaluating candidate drought-resistance genes in soybean.

## Materials and methods

### Plant materials

Soybean (*Glycine max* [L.] Merr. cv. Williams 82) seeds were germinated on moistened vermiculite at 25°C overnight, and the seed coats were removed prior to bombardment with ALSV RNA (see below). After bombardment, the soybean seedlings were incubated on moistened filter paper at 18°C in darkness for 2 days and transplanted to pots (9.5 cm diameter) filled with 250 g of culture soil (Tsuchitaro: Sumitomo Forestry, Ama, Aichi, Japan), with a weight water content of approximately 50%. The pots were placed in a growth chamber at 25°C with 50% humidity and 400 ppm CO_2_ under a 12 h light/12 h dark photoperiod and a light intensity of 150 μmol photons m^-2^ s^-1^. The soybean growth stages such as V4 and V5 were determined as described previously [39]. For virus propagation, *Chenopodium quinoa* plants were grown in a greenhouse or growth chamber at 25°C, as described previously [40]. *C*. *quinoa* was germinated on Sakata Super Mix A soil (Sakata Seed, Yokohama, Kanagawa, Japan) and planted in culture soil (Tsuchitaro: Sumitomo Forestry or Gacchirikun N-120: Tokita Seed, Saitama, Japan).

### RNA isolation and RT-PCR analysis

Total RNA was isolated from *C*. *quinoa* and soybean using RNAiso Plus (Takara Bio, Otsu, Shiga, Japan) according to the manufacturer’s instructions. Total RNA was pre-treated with RQ1 RNase-free DNase (Promega, Madison, WI, USA) and complementary DNA (cDNA) was synthesized using PrimeScript RT Master Mix (Takara Bio). Quantitative reverse-transcription (qRT) polymerase chain reaction (PCR) was performed using an ABI7500 Real-Time PCR system (Life Technologies, Grand Island, NY, USA), and SYBR Premix Ex Taq (Takara Bio). Virus RNA was detected by RT-PCR analysis using GoTaq Green Master Mix (Promega). The specific oligonucleotides used to amplify each gene are described in S1 Table. Statistical analysis was performed using StatPlus (v6 for Mac, AnalystSoft Inc.).

### DNA constructs for VIGS analysis

To create the ALSV-RNA2 vectors for VIGS analyses, fragments containing nucleotides 268–567 and 961–1,260 from *GmERA1B* (*Glyma13g23780*) and 274–573 from *GmPDS* (*Glycine max phytoene desaturase*, *Glyma18g00720*) were amplified from soybean cDNA by PCR using the specific primer pairs GmERA1-F01/R01, GmERA1-F02/R02, and GmPDS-F274/R573, respectively (S1 Fig, S1 Table). The amplified DNA fragments were cloned in-frame into the *Xho*I/*Bam*HI site of pEALSR2 (ALSV-RNA2 plasmid) [24], generating pEALSR2-GmERA1N, pEALSR2-GmERA1C, and pEALSR2-GmPDS, respectively. *C*. *quinoa* was inoculated with either of these ALSV-RNA2 constructs along with pEALSR1 (the ALSV-RNA1 plasmid) by mechanical inoculation [24], and the resulting viruses were designated ALSV-GmERA1N, ALSV-GmERA1C, and ALSV-GmPDS, respectively. ALSV-GmERA1N and ALSV-GmERA1C were designed to target both *GmERA1A* and *GmERA1B* due to their high level of nucleotide sequence similarity (S1A Fig). *GmPDS* encodes a key enzyme required for carotenoid biosynthesis, and *GmPDS* knockout mutants have an albino phenotype that can readily be detected; therefore, ALSV-GmPDS was used as a visible VIGS indicator [23]. Wild-type ALSV derived from *C*. *quinoa* plants inoculated with pEALSR1 and pEALSR2 was used as a vector control (ALSV-VC). All plasmid DNAs used for VIGS analysis were prepared using a QIAGEN Plasmid Midi Kit (Qiagen, Hilden, Germany).

### Virus inoculation

*C*. *quinoa* and soybean plants were inoculated with ALSV as described [19, 23, 41]. The plasmids for ALSV-RNA1 and RNA2 were mixed in equal amounts, and the DNA solution was mechanically inoculated onto the true leaves of *C*. *quinoa* using carborundum. Reverse osmosis (RO) water was used for mock inoculation. The inoculated *C*. *quinoa* plants were grown for two to three weeks. The upper leaves were sampled and ground in three volumes of extraction buffer (0.1 M Tris-HCl, pH 8.0, 0.1 M NaCl, 5 mM MgCl_2_ [23]). Debris was precipitated by centrifugation, and the supernatants were used for secondary inoculation of *C*. *quinoa*. After two to three weeks, the upper leaves were sampled and stored at -80°C. Total RNA, extracted from the upper leaves of *C*. *quinoa* plants subjected to a second inoculation, was used as an inoculum for soybean. Biolistic inoculation of germinated soybean seeds was performed using a PDS-1000/He Particle Delivery System (Bio-Rad, Hercules, CA, USA) and 1.0 Micron Gold Microcarrier (Bio-Rad) particles that had been coated with total RNA from *C*. *quinoa* as described [23]. Seven to ten germinated soybean seeds were placed onto a Petri dish and bombarded twice at 1,100 psi. Approximately 7 μg of total RNA was used per shot.

### Measurement of physiological changes in VIGS plants

Water loss rates were measured as described [42], with minor modifications. A leaflet from the fourth or fifth trifoliate leaves of a virus-infected soybean plant was excised and fresh weight was measured over time. Thermal images of leaves were taken using an FLIR E60 thermal imaging camera according to the manufacturer’s protocol (FLIR Systems, Wilsonville, OR, USA). The H_2_O conductance rates and leaf temperature were measured with a LI-6400XT portable photosynthesis system (Li-Cor Bioscience, Lincoln, NE, USA). The CO_2_ concentration of the input flow, chamber block temperature, and level of the LED light source were set at 400 μmol mol^-1^, 25°C, and 500 μmol m^-2^ s^-1^, respectively. A fourth or fifth trifoliate leaflet was placed in the leaf chamber of the LI-6400XT system, and time 0 was set after the measured values stabilized. After a measurement was taken at time 0, the leaflet was detached and the data were collected every 30 s. Stomatal apertures were measured as described [31, 42], with minor modifications. Leaf disks of 1 cm^2^ were excised from the fourth trifoliate leaves of ALSV-infected soybean plants, incubated for 4 h in 20 mM KCl, 1 mM CaCl_2_, 5 mM MES-KOH, pH 6.15, and 0.003% Silwet-77, and treated with or without 10 μM ABA for 4 h under the light condition. Photographs of guard cells were taken through a color laser three-dimensional profile microscope (Keyence, Osaka, Japan). Stomatal apertures were measured using ImageJ software (http://imagej.nih.gov/ij/). Drought tolerance assays were conducted as described [42] with minor modifications. Water was withheld from whole plants at growth stage V6 [39] for 3 days. Six independent trials were carried out, including three blind tests.

## Results

### Sequence analysis of *GmERA1* genes

*Arabidopsis ERA1* plays an important role in ABA signaling [29–31] and is a promising candidate gene for genetic manipulation of drought stress tolerance in *Arabidopsis* and canola plants [35, 36]. Although drought is considered to be a major problem in soybean production worldwide [43–45], *ERA1* homologs in soybean have not been reported to date. Therefore, we searched the Soybean Knowledge Base (SoyKB, http://soykb.org/) using BlastP program and the amino acid sequence of *Arabidopsis* ERA1 as query. Two soybean genes, *Glyma06g19740* and *Glyma13g23780*, were identified as homologs of *Arabidopsis ERA1* and named *GmERA1A* and *GmERA1B*, respectively (S1 and S2 Figs, S2 Table). S2 Fig shows the sequence alignment of ERA1 proteins from soybean and *Arabidopsis*. GmERA1A and GmERA1B share 56.0% and 56.3% amino acid sequence identity with *Arabidopsis* ERA1, respectively. GmERA1A shares 92.9% amino acid sequence identity with GmERA1B. These data are consistent with the previous finding that soybean is a partially diploidized tetraploid [12]. Although these proteins have high levels of amino acid sequence identity, the N-terminus of *Arabidopsis* ERA1 is longer than that of GmERA1A or GmERA1B (S2 Fig). Five prenyltransferase domains are well-conserved in the ERA proteins (S2 Fig). An analysis of public databases in the Soybean and Arabidopsis eFP Browsers (http://bar.utoronto.ca/)) showed that both *GmERA1A* and *GmERA1B*, like *Arabidopsis ERA1*, are expressed in various tissues (S3 Fig).

### VIGS of *GmERA1* genes in soybean

We next sought to analyze the function of *GmERA1* genes by manipulating their expression in soybean plants. Since the transformation efficiency of soybean is much lower than that of *Arabidopsis* and rice [13, 46, 47], we used VIGS to down-regulate the *GmERA1* genes in wild-type soybean plants. To date, BPMV, CMV, and ALSV vectors have been used for VIGS in soybean [17, 18, 19]. Because soybean is not a natural host of ALSV, in contrast to BPMV and CMV, we used an ALSV vector in the current study to minimize potential interference effects imposed by naturally compatible virus-host interactions on abiotic stress responses. To examine the effect of loss-of-function of *GmERA1* genes in soybean plants, we performed ALSV-mediated VIGS in the sequenced model soybean cultivar Williams 82. We obtained ALSV-GmERA1N, ALSV-GmERA1C, ALSV-GmPDS, and ALSV-VC through propagating the virus in *C*. *quinoa* plants inoculated with the ALSV-RNA1 cDNA plasmid combined with ALSV-RNA2 VIGS vectors either harboring or not a specific 300-bp trigger sequence required for VIGS (S1 and S4 Figs). ALSV-GmERA1N and ALSV-GmERA1C were designed to target both *GmERA1A* and *GmERA1B* due to their high level of nucleotide sequence similarity (95.3%) (S1A Fig). The empty vector (ALSV-VC) and RO water (Mock) were used as negative controls. ALSV-GmPDS was used as a visible VIGS indicator [23].

Mild mosaic symptoms appeared in unifoliate leaves and were still visible in the third trifoliate leaves of soybean plants infected with ALSV-VC (S5A Fig), but symptoms were nearly absent in the fourth trifoliate leaves (S5B Fig). By contrast, in plants infected with ALSV-GmPDS, an entire photobleaching phenotype was observed in the fourth trifoliate leaves and subsequent ones (S5B Fig). These observations are similar to those reported previously [19, 48], indicating that ALSV-mediated VIGS can be observed throughout the leaves after the fourth trifoliate stage of development.

**Fig 1:**
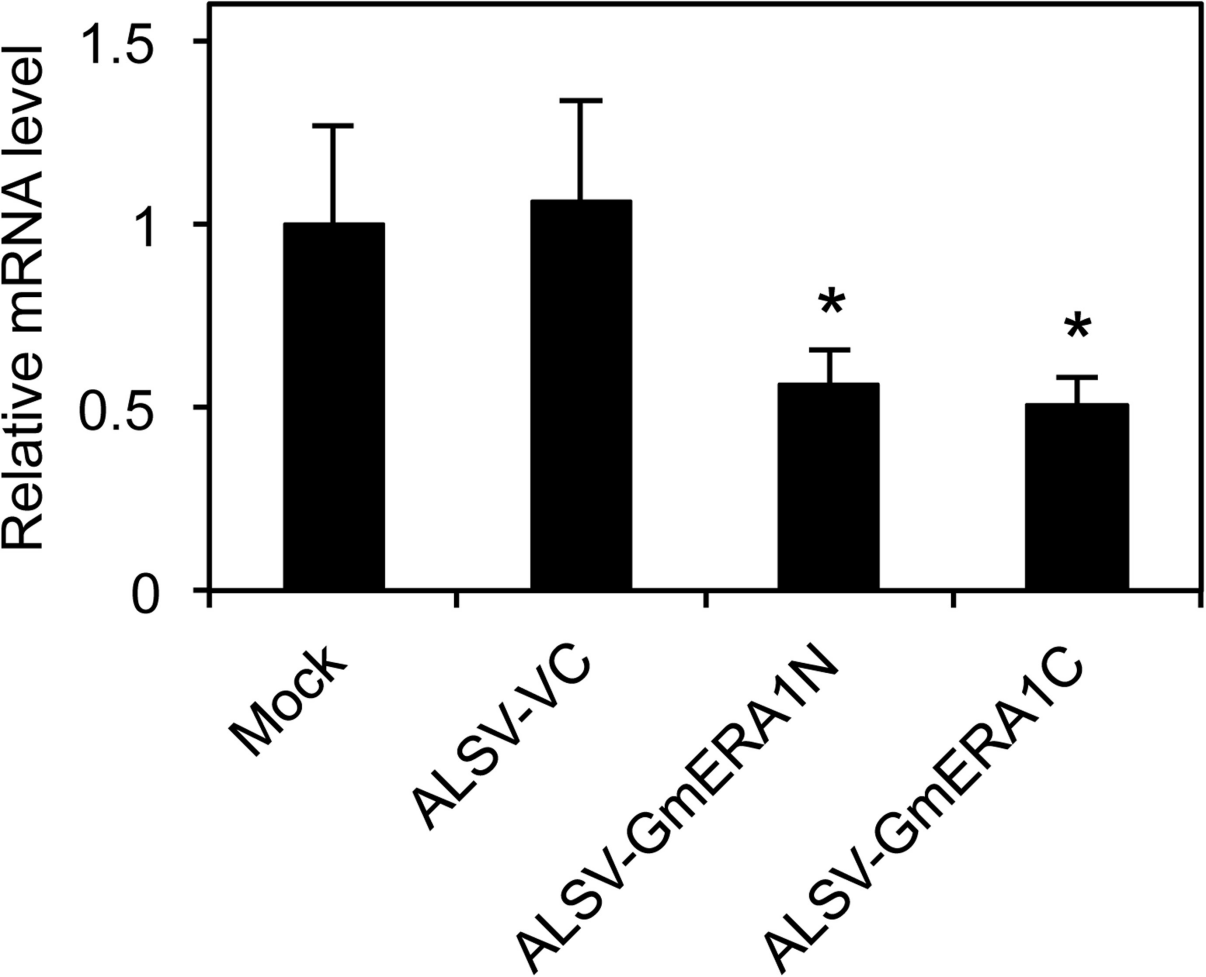
ALSV infection induces VIGS of *GmERA1s* in soybean. Silencing efficiency was evaluated by examining the expression of *GmERA1s* using quantitative RT-PCR and total RNAs derived from the fourth trifoliate leaves of soybean plants inoculated with the indicated inoculums. A conserved region in the two *GmERA1* genes was amplified by qRT-PCR. Relative values are presented as mean ± SD (*n* = 3) normalized to the expression of *GmACT11* (*Glyma15g05570*) as a control. Asterisks indicate a significant difference from ALSV-VC (empty vector) by *t*-test (*P < 0.05).

We performed qRT-PCR analysis to analyze the silencing efficiency of *GmERA1* genes in the fourth trifoliate leaves (Fig 1) using a primer set that could amplify both *GmERA1s* (S1 Fig). In the fourth trifoliate leaves of plants inoculated with ALSV-GmERA1N or ALSV-GmERA1C, *GmERA1A* and *GmERA1B* were expressed at levels half of those in control plants inoculated with ALSV-VC or mock inoculum (Fig 1, S6 Fig), suggesting that the endogenous *GmERA1A* and *GmERA1B* genes were successfully down-regulated in plants inoculated with ALSV-GmERA1N and ALSV-GmERA1C. The leaflet size of the fourth trifoliate leaves and the plant height were similar between soybean plants inoculated with ALSV-GmERA1N or ALSV-GmERA1C and plants inoculated with ALSV-VC or mock inoculum (S7 Fig).

### Down-regulation of *GmERA1s* reduces water loss and gas exchange under water limitation

**Fig 2:**
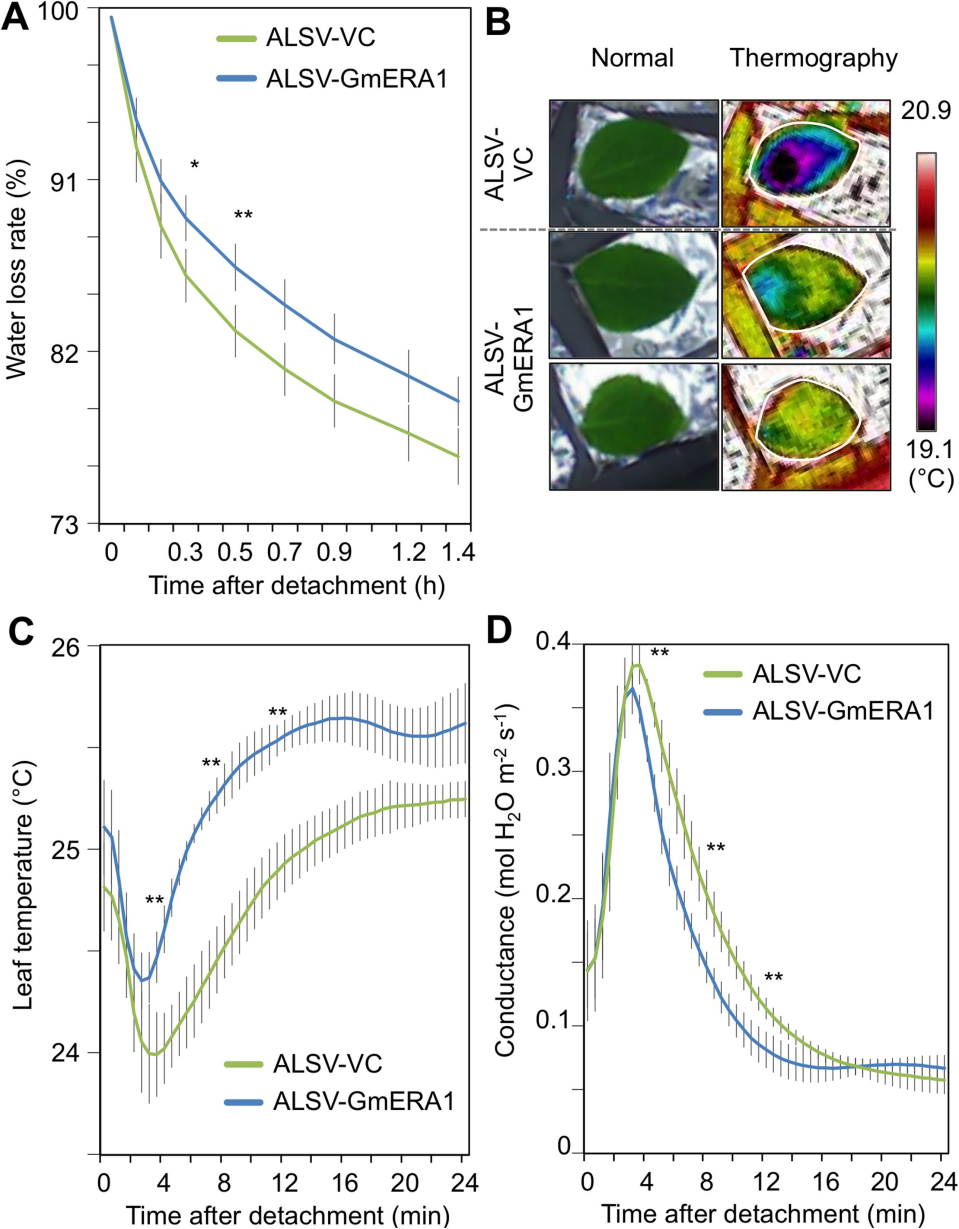
*GmERA1*-down-regulated leaves show reduced water loss rates and stomatal conductance. (A) Rates of water loss in the fourth or fifth trifoliate leaves of soybean plants inoculated with ALSV vectors. Each data point represents the mean of duplicate measurements (*n* = 3 for ALSV-VC and *n* = 6 for ALSV-GmERA1). Bars indicate SD. Asterisks at 0.3 and 0.5 h indicate a significant difference between ALSV-VC and ALSV-GmERA1 by *t*-test (*P < 0.05, **P < 0.01). (B) The surface temperature of detached fourth or fifth trifoliate leaflets of soybean plants inoculated with ALSV vectors was measured using thermography during the water-loss experiment shown in (A). Thermal images and photographs were taken 11 min after detachment. (C and D) Evaluation of leaf temperature (C) and stomatal conductance (D) in detached fourth or fifth trifoliate leaflets of soybean plants inoculated with ALSV vectors using a LI-6400XT portable photosynthesis system. Values are presented as mean ± SD (*n* = 3 for ALSV-VC and *n* = 4 for ALSV-GmERA1), and asterisks at 4, 8, and 12 min indicate a significant difference between ALSV-VC and ALSV-GmERA1 by *t*-test (**P < 0.01).

*ERA1*, which is expressed in guard cells, regulates the transpiration rate in *Arabidopsis* by modulating ABA-mediated stomatal responses [29, 31]. We therefore measured water loss in the leaves of *GmERA1*-down-regulated plants to evaluate their response to drought stress. The water loss rate was lower in the detached fourth or fifth trifoliate leaves of soybean plants inoculated with ALSV-GmERA1s than in those of plants inoculated with ALSV-VC (Fig 2A), suggesting that transpiration from the leaves of *GmERA1*-down-regulated plants was reduced under limited water conditions. This result is consistent with our infrared thermography observation that the leaf temperature of detached fourth trifoliate leaflets of plants inoculated with ALSV-GmERA1s was higher than that of detached fourth trifoliate leaflets of plants inoculated with ALSV-VC (Fig 2B). To confirm these results, we analyzed the leaf surface temperature over time using a LI-6400XT portable photosynthesis system. At 3 or more min after detachment, the surface temperature of detached fourth or fifth trifoliate leaflets of *GmERA1*-down-regulated plants was ~1°C higher than that of control plants (Fig 2C). Furthermore, using the LI-6400XT system, we measured the rate of gas exchange in the detached *GmERA1*-down-regulated leaflets over time. At 4 to 16 min after detachment, the water-stressed fourth or fifth trifoliate leaves of *GmERA1*-down-regulated plants exhibited statistically significant reductions in stomatal conductance compared to that of control plants inoculated with ALSV-VC empty viral vector, suggesting that down-regulation of *GmERA1s* reduces gas exchange under water-limiting conditions (Fig 2D). These results support the notion that down-regulation of *GmERA1s* reduces water loss from water-stressed soybean leaves through its influence on stomatal regulation.

### Down-regulation of *GmERA1s* enhances the stomatal response to ABA in soybean leaves

**Fig 3:**
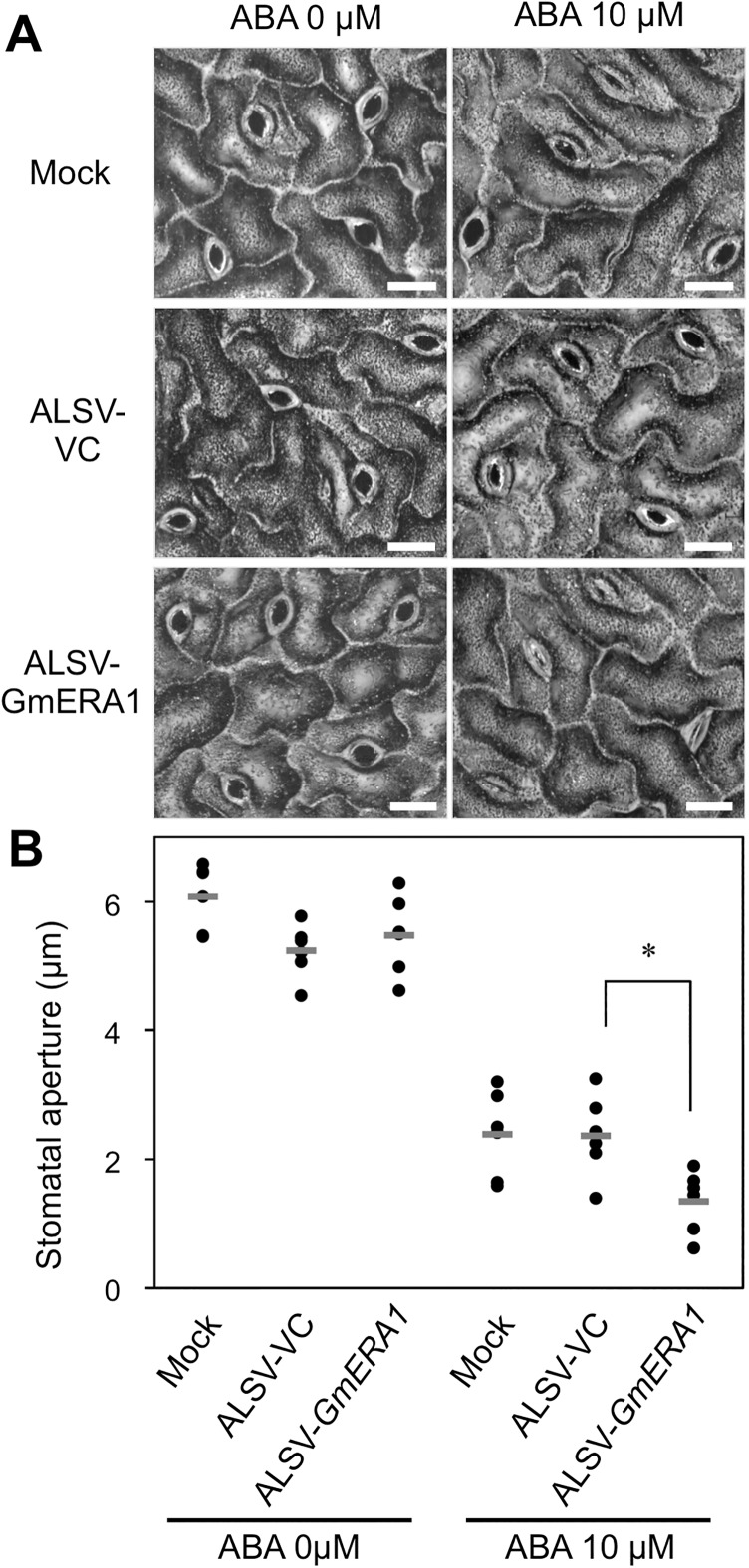
Down-regulation of *GmERA1s* enhances the stomatal closure response to ABA. Leaf disks from the fourth trifoliate leaves of ALSV-infected soybean plants were treated with or without ABA for 4 h. (A) Representative photographs of leaflets after ABA treatment (scale bars = 20 μm). (B) Stomatal aperture size data (shown as dots) from six independent measurements (≥60 stomata each). Bars indicate mean values. *P = 0.010, by *t*-test.

ERA1 is a well-known negative regulator of ABA responses in *Arabidopsis* [30]. To examine whether *GmERA1s*-down-regulation affects stomatal responses to ABA, we analyzed stomatal apertures in the leaves of *GmERA1*-down-regulated plants treated with or without ABA. In the absence of ABA, no marked difference in stomatal aperture in the fourth trifoliate leaves was detected between *GmERA1*-down-regulated and control plants. However, in the presence of 10 μM ABA, the stomatal aperture in these leaves was significantly narrower in plants inoculated with ALSV-GmERA1s than in ALSV-VC or mock-inoculated plants (Fig 3), indicating that down-regulation of *GmERA1s* enhances the stomatal closure response to ABA. This result also suggests that GmERA1s function as negative regulators of ABA responses, as does *Arabidopsis* ERA1. Taken together, these data support the notion that altered ABA-mediated stomatal closure contributes to the reduced water loss rate of *GmERA1*-down-regulated leaves under water-limiting conditions.

### *GmERA1*-down-regulated plants exhibit enhanced resistance to water-deficit stress

**Fig 4:**
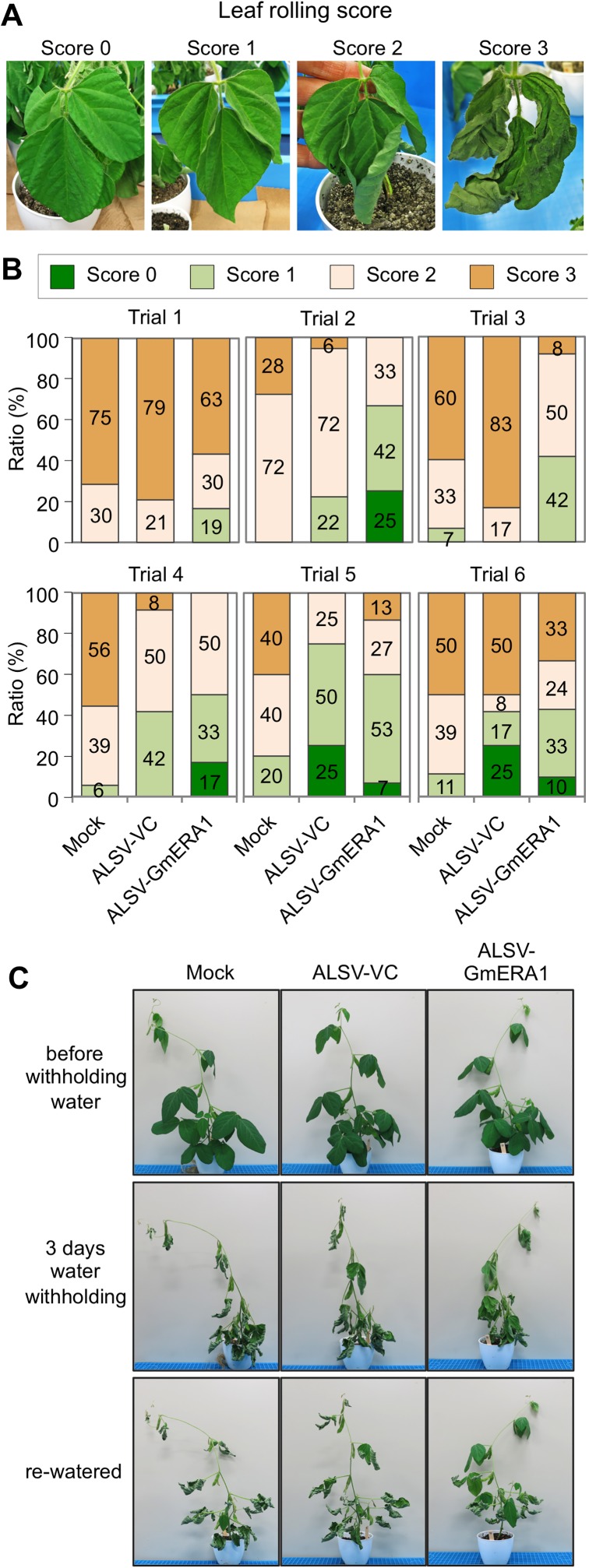
Evaluation of drought resistance in ALSV-GmERA1-infected soybean plants. Watering was withheld from plants at the V6 growth stage for 3 days. The drought status of the fourth to sixth trifoliate leaves was scored from 0 to 4 as follows: 0, no change; 1, wilting; 2, rolling; and 3, severe wilting. (A) Representative photographs of leaves with various scores. The leaves were scored at several time points after water withholding. (B) Leaf rolling score before rehydration (72–75 h after water withholding). The trial was repeated independently six times. (C) Plants were re-watered at 3 days after water withholding. Representative plants at one day after rehydration are shown.

We demonstrated that the *GmERA1*-down-regulated leaves displayed reduced water loss and gas exchange through ABA-mediated stomatal regulation compared to the vector control plants. Next, we examined whether whole *GmERA1*-down-regulated soybean plants exhibit enhanced drought tolerance. When watering was withheld from soybean plants at the V6 growth stage [39] for 24 h, the leaf temperature was higher in plants inoculated with ALSV-GmERA1N and ALSV-GmERA1C than in mock- or ALSV-VC-inoculated plants (S8 Fig). However, during the first 24 h of dehydration, no wilting was observed among the plants (S8 Fig). We then scored leaf rolling in the fourth to sixth trifoliate leaves after approximately 60 h of dehydration stress treatment in six independent experiments (Fig 4A). At 60 h post-treatment, the leaf rolling scores of the plants were similar (S9 Fig). However, after 72–75 h of dehydration stress treatment, although ambient humidity could not be controlled under our experimental conditions, in five out of six independent experiments, the ratio of severely wilted leaves to total leaves (score 3) was lower in *GmERA1*-down-regulated soybean plants than in the control, indicating that in many cases, the *GmERA1*-down-regulated soybean plants exhibited less wilting than the control (Fig 4B). At one day after rehydration, the survival rates of the plants in each experiment were quite variable (Fig 4C, S3 Table). Nevertheless, taking all six independent experiments into account, approximately 60% of the *GmERA1*-down-regulated soybean plants survived, whereas only approximately 30% of the control plants survived (Fig 4C, S3 Table). Overall, the *GmERA1*-down-regulated soybean plants survived dehydration stress better than did the vector control plants in terms of wilting, supporting the view that *GmERA1*-down-regulated soybean plants exhibit enhanced resistance to water-deficit conditions.

## Discussion

Our data show that *GmERA1*-down-regulated leaves displayed reduced water loss rates under water-limiting conditions through ABA-mediated stomatal regulation and, consequently, that these plants exhibited enhanced resistance to water deficit conditions, consistent with previous studies of plants with similar levels of down-regulation of *ERA1* homologs [35–37]. These findings demonstrate that *GmERA1s* function as negative regulators of ABA signaling in guard cells in soybean. Therefore, *GmERA1s* appear to play an important role in the dehydration stress response in soybean, as does *ERA1* in *Arabidopsis* [29–31, 35]. Our data support the hypothesis that *ERA1* genes can be downregulated to increase sensitivity to ABA, thereby leading to more rapid ABA-induced stomatal closure under water-limiting conditions. Thus, our study suggests a useful strategy for enhancing drought resistance in soybean. Plants harboring mutations in *GmERA1s* could be readily identified through high-throughput methods, such as targeting-induced local lesions in genomes (TILLING), high-resolution melting (HRM), and indexed amplicon sequencing by next generation sequencing (NGS), taking advantage of high-density mutant resources that have already been developed in soybean [49–51].

This is the first report describing the successful use of VIGS in soybean to functionally characterize genes involved in drought stress resistance and ABA signaling. As shown in this and previous work [15, 52], the greatest merit of the VIGS system is that it allows gene functions to be characterized easily and rapidly without the need to generate transgenic plants or to perform large-scale forward mutagenesis screening. However, this and previous studies also indicate that the leaf positions and growth stage in which gene functions can be analyzed are limited. Indeed, we mainly analyzed the fourth or fifth trifoliate leaves based on the spread of silencing symptoms caused by ALSV-GmPDS in soybean plants (Figs 1–3, S5 Fig). More variable results were obtained in the drought resistance tests using whole plants instead of isolated leaves (Figs 2–4, S3 Table), which is consistent with previous reports showing that statistically significant results could not be obtained in VIGS experiments yielding plants with enhanced drought resistance at the whole-plant level [37, 53]. Thus, the weakness of the VIGS system is the instability in the extent of temporal and spatial gene silencing due to variations in virus infection caused by environmental factors and plant growth, as described previously [47, 54]. Improving virus infection rates and stable spread within an infected plant would increase the utility of the VIGS system.

Nonetheless, the weaknesses of the VIGS system described above can also be perceived as strengths. Although the growth of *ERA1*-repressed *Arabidopsis* plants is retarded [55–58], under our experimental conditions, no significant negative effect on plant growth was observed in the *GmERA1*-down-regulated plants (S7 Fig). As specific leaf positions and growth stages (e.g., the fourth trifoliate leaves at the V4 growth stage) are more readily recognized in soybean than in other staple crops such as wheat and *Solanum lycopersicum* (tomato) [39], the ALSV-VIGS system is ideal for validating gene function in soybean. Thus, our findings demonstrate that the ALSV-VIGS system is a useful tool for validating candidate genes without the need for generating transgenic plants; however, the selected candidate genes will need to be evaluated using appropriate mutant or transgenic plants to assess the impact on physiological parameters, such as soil water contents, before applying this technology in the field. Therefore, the ALSV-VIGS system, combined with recent methods for screening for desired mutations from high-density mutant resources and the CRISPR/CAS9-mediated introduction of desired mutations, could contribute to next-generation molecular breeding strategies to improve drought resistance in soybean.

## Supporting information

S1 Fig Alignment of *GmERA1A* and *GmERA1B* nucleotide sequences and map of the trigger sequences of the ALSV vectors. (A) Sequence alignment of two *AtERA1* homologs from soybean. The coding sequences of *Glyma06g19740* (*GmERA1A*) and *Glyma13g23780* (*GmERA1B*) were aligned using ClustalW2. Different bases between two genes are highlighted on the *Glyma06g19740* sequence. Two VIGS trigger sequences on *Glyma13g23780* are shown in gray. The sequences used for the alignment are shown in S2 Table. (B) Schematic diagram of the trigger sequences in the ALSV-GmERA1N and ALSV-GmERA1C viruses. Two trigger sequences were designed in the CDS of *Glyma13g23780* using in-frame cloning. The region amplified in the qRT-PCR analysis is also shown. The primer set used for qRT-PCR analysis could amplify both homologs, *Glyma13g23780* and *Glyma06g19740*.
(PDF)

S2 Fig Comparison of amino acid sequences of Glyma06g19740, Glyma13g23780, and AtERA1 (At5g40280). Conserved amino acids are marked with asterisks (*); conserved and semi-conserved substitutions of amino acids are marked with a colon (:) and a period (.), respectively. Amino acid sequences were aligned using ClustalW2 with default settings. The five prenyltransferase domains are indicated with magenta boxes. The sequences used for the alignment are shown in S2 Table.
(PDF)

S3 Fig Expression status of *GmERA1A* and *GmERA1B* in soybean plants. mRNA expression profiles were obtained from the (A) Soybean eFP Browser and (B) Arabidopsis eFP Browser (http://bar.utoronto.ca/welcome.htm). (A) Data for the ‘Relative’ expression level of *GmERA1A* (*Glyma06g19740*) and *GmERA1B* (*Glyma13g23780*) were obtained. (B) Data for the ‘Relative’ expression level of *AtERA1* (*At5g40280*) were obtained from the data source of ‘Developmental Map’.
(PDF)

S4 Fig Inoculation of *Chenopodium quinoa* plants with recombinant ALSV plasmids. (A) Mosaic symptoms, indicating virus infection, appeared in the upper leaves of *C*. *quinoa* inoculated with ALSV plasmids approximately one week after inoculation. The photograph was taken 3 weeks after inoculation. *C*. *quinoa* leaves with mosaic symptoms (encircled with magenta dashed lines) were used for inoculum preparation. The inoculum was used for secondary inoculation of another *C*. *quinoa* plant. (B) Detection of RNA1 and RNA2 for ALSV by RT-PCR. The cDNA samples were prepared from *C*. *quinoa* leaves exhibiting mosaic symptoms. Equal amounts of total RNA (used for cDNA synthesis) were loaded as a control. Bands with asterisks (*) in ALSV-RNA2 indicate the position of the original size of each virus construct.
(PDF)

S5 Fig Inoculation of soybean plants with recombinant ALSVs and induction of virus-induced gene silencing. (A) Mosaic symptoms in young soybean plants infected with ALSV. Mosaic symptoms were observed in unifoliate (left photograph) and early first-to-second trifoliate (right photograph) leaves. Representative mock-inoculated and ALSV-VC (vector control)-inoculated plants are shown. Leaves with mosaic symptoms are encircled with dashed lines. (B) Lack of mosaic symptoms and complete expansion of ALSV infection in fourth trifoliate leaves. Appreciable mosaic symptoms appeared in the third trifoliate leaves, although almost no symptoms were present in the fourth trifoliate leaves (left photograph; ALSV-VC). Bleaching symptoms caused by VIGS in soybean plants infected with ALSV-GmPDS appear to be complete in the fourth trifoliate leaves (right photograph; ALSV-GmPDS). Magenta and white dashed lines indicate fourth and third trifoliate leaves, respectively.
(PDF)

S6 Fig Expression analysis of *GmERA1A* and *GmERA1B* in ASLV-infected leaves. Total RNA samples were prepared from an attached or a 4.5 h-detached leaflet in ALSV-VC or ALSV-GmERA1N-infected soybean. (A) Both *GmERA1A* and *GmERA1B* were amplified with the same primer set (RT-F1 and RT-R1). (B) *GmERA1A* and (C) *GmERA1B* were amplified with gene-specific primer sets (RT-F2 and RT-R2 for *GmERA1A*; and RT-F3 and RT-R3 for *GmERA1B*). Relative values are presented as mean ± SD (*n* = 4) normalized to the expression of *GmACT11* as a control. Asterisks (*) denote a significant difference from the ALSV-VC (empty vector) control by *t*-test (*P* < 0.05).
(PDF)

S7 Fig Comparison of plant sizes among soybean plants infected with ALSVs. (A) Plants photographed 5 weeks after inoculation with ALSV. Arrows indicate the fourth trifoliate leaves in each plant. (B and C) The size of the fourth trifoliate leaf (B) and the plant height and length of the stem from the first to the fourth internode (C) were scored 5 weeks after inoculation with ALSV (*n* = 4, mean ± SE). No significant difference in plant size was detected between plants inoculated with ALSV-VC and ASLV-GmERA1 at growth stages V4–V6.
(PDF)

S8 Fig Changes in the surface temperatures of whole plants during the water withholding test. Watering was withheld from potted five-week-old plants at the V6 growth stage. The surface temperature of whole plants was measured at 24 h after water withholding. A representative photograph is shown.
(PDF)

S9 Fig Leaf rolling scores for ALSV-infected soybean plants subjected to water withholding. During the water-withholding test, the drought status of the fourth to sixth trifoliate leaves was scored as described in Fig 4. Leaf drought scores at 55–62 h after water withholding are shown for six independent trials.
(PDF)

S1 Table Oligonucleotide primers used in this study. (XLSX)

S2 Table ERA1 sequences used for the alignment. (XLSX)

S3 Table Survival rates in the water-withholding test. (XLSX)
