# Supplementary material for: Virus-induced down-regulation of GmERA1A and GmERA1B genes enhances the stomatal response to abscisic acid and drought resistance in soybean
Source: PLoS One. 2017 Apr 18;12(4):e0175650. doi: 10.1371/journal.pone.0175650 (PMC5395220; doi:10.1371/journal.pone.0175650)
Supplement: S1 Fig — (A) Sequence alignment of two AtERA1 homologs from soybean. The coding sequences of Glyma06g19740 (GmERA1A) and Glyma13g23780 (GmERA1B) were aligned using ClustalW2. Different bases between two genes are highlighted on the Glyma06g19740 sequence. Two VIGS trigger sequences on Glyma13g23780 are shown in gray. The sequences used for the alignment are shown in S2 Table. (B) Schematic diagram of the trigger sequences in the ALSV-GmERA1N and ALSV-GmERA1C viruses. Two trigger sequences were designed in the CDS of Glyma13g23780 using in-frame cloning. The region amplified in the qRT-PCR analysis is also shown. The primer set used for qRT-PCR analysis could amplify both homologs, Glyma13g23780 and Glyma06g19740. (PDF) [file pone.0175650.s001.pdf]

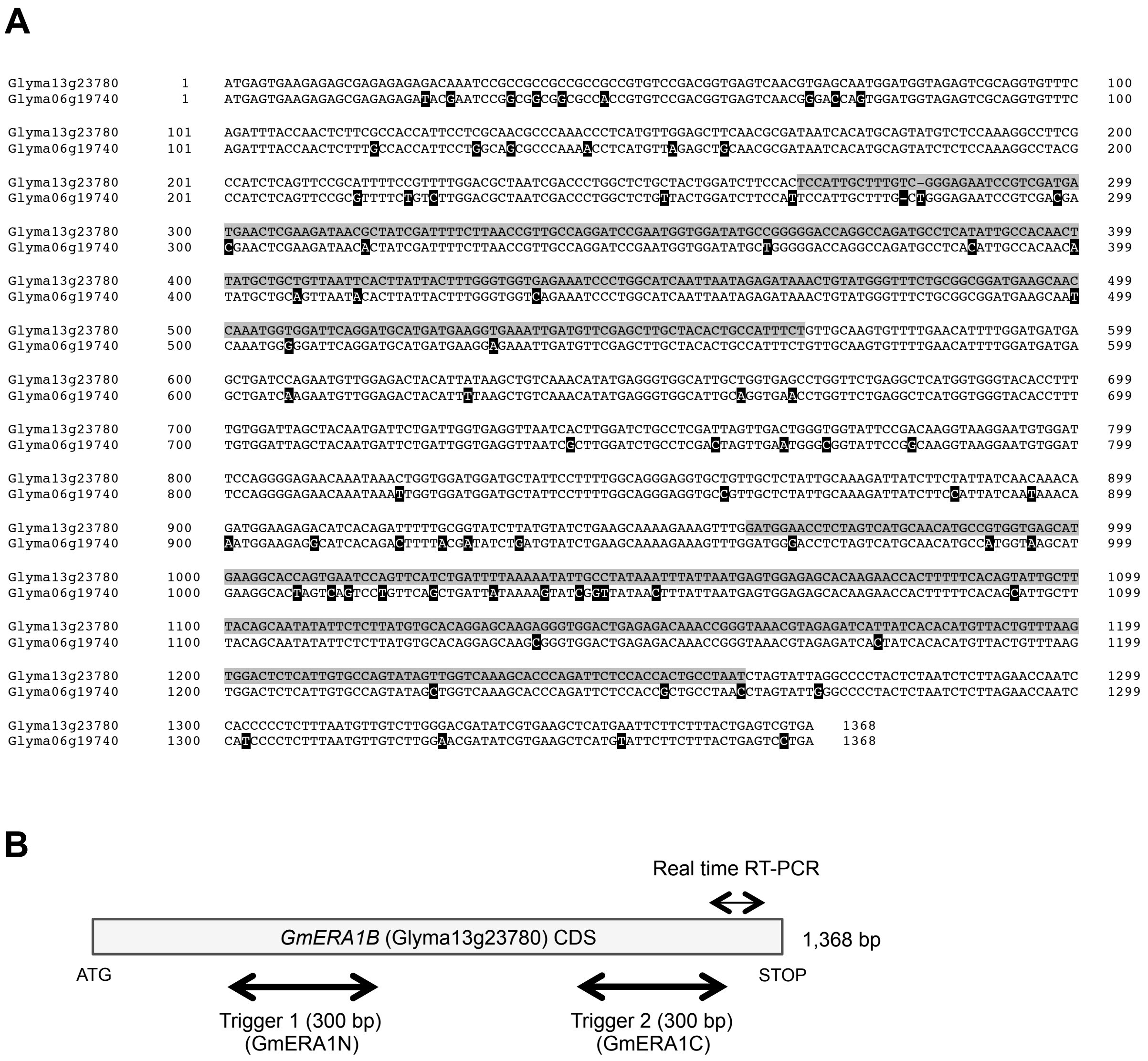

S1 Fig. Alignment of *GmERA1A* and *GmERA1B* nucleotide sequences and map of the trigger sequences of the ALSV vectors.
