# Supplementary material for: Virus-induced down-regulation of GmERA1A and GmERA1B genes enhances the stomatal response to abscisic acid and drought resistance in soybean
Source: PLoS One. 2017 Apr 18;12(4):e0175650. doi: 10.1371/journal.pone.0175650 (PMC5395220; doi:10.1371/journal.pone.0175650)
Supplement: S2 Fig — Conserved amino acids are marked with asterisks (*); conserved and semi-conserved substitutions of amino acids are marked with a colon (:) and a period (.), respectively. Amino acid sequences were aligned using ClustalW2 with default settings. The five prenyltransferase domains are indicated with magenta boxes. The sequences used for the alignment are shown in S2 Table. (PDF) [file pone.0175650.s002.pdf]

```
Glyma06g19740       -------------------------MSEESERDTNPA---AAPPCPTVSQRDQWMVESQV     32
Glyma13g23780       -------------------------MSEESERETNPP---PPPPCPTVSQREQWMVESQV     32
AtERA1/At5g40280    MPVVTRLIRLKCVGLRLDRSGLNRRICHGGHGESTRRRVMEELSSLTVSQREQFLVENDV     60
                                             :.. .. ::.        .. *****:*::**.:*

Glyma06g19740       FQIYQLFATIPGSAQNLMLELQRDNHMQYLSKGLRHLSSAFSVLDANRPWLCYWIFHSIA     92
Glyma13g23780       FQIYQLFATIPRNAQTLMLELQRDNHMQYVSKGLRHLSSAFSVLDANRPWLCYWIFHSIA     92
AtERA1/At5g40280    FGIYNYFDASDVSTQKYMMEIQRDKQLDYLMKGLRQLGPQFSSLDANRPWLCYWILHSIA     120
                    * **: * :   .:*. *:*:***::::*: ****:*.. ** ***********:****

Glyma06g19740       LLGESVDDELEDNTIDFLNRCQDPNGGYAGGPGQMPHIATTYAAVNTLITLGGQKSLASI     152
Glyma13g23780       LSGESVDDELEDNAIDFLNRCQDPNGGYAGGPGQMPHIATTYAAVNSLITLGGEKSLASI     152
AtERA1/At5g40280    LLGETVDDELESNAIDFLGRCQGSEGGYGGGPGQLPHLATTYAAVNALVTLGGDKALSSI     180
                    * **:*******.*:****.***..:***.*****:**:********:*:****:*:*:**

Glyma06g19740       NRDKLYGFLRRMKQSNGGFRMHDEGEIDVRACYTAISVASVLNILDDELIKNVGDYILSC     212
Glyma13g23780       NRDKLYGFLRRMKQPNGGFRMHDEGEIDVRACYTAISVASVLNILDDELIQNVGDYIISC     212
AtERA1/At5g40280    NREKMSCFLRRMKDTSGGFRMHDMGEMDVRACYTAISVASILNIMDDELTQGLGDYILSC     240
                    **:*:  ******:..******* **:*************:***:**** :.:****:**

Glyma06g19740       QTYEGGIAGEPGSEAHGGYTFCGLATMILIGEVNRLDLPRLVEWAVFRQGKECGFQGRTN     272
Glyma13g23780       QTYEGGIAGEPGSEAHGGYTFCGLATMILIGEVNHLDLPRLVDWVVFRQGKECGFQGRTN     272
AtERA1/At5g40280    QTYEGGIGGEPGSEAHGGYTYCGLAAMILINEVDRLNLDSLMNWAVHRQGVEMGFQGRTN     300
                    *******.************:****:****.**::*:*   *::*.*.*** * *******

Glyma06g19740       KLVDGCYSFWQGGAVALLQRLSSIINKQMEEASQTFTISDVSEAKESLDGTSSHATCHGK     332
Glyma13g23780       KLVDGCYSFWQGGAVALLQRLSSIINKQMEETSQIFAVSYVSEAKESLDGTSSHATCRGE     332
AtERA1/At5g40280    KLVDGCYTFWQAAPCVLLQRLYSTNDHDVHGSSHISEGTNEEHHAHDEDDLEDSDDDDDS     360
                    *******:***... .***** *  ::::. :*:     :  ..  .. *._..     ..

Glyma06g19740       HEGTSQSCSADYKSIGYNFINEWRAQEPLFHSIALQQYILLCAQEQAGGLRDKPGKRRDH     392
Glyma13g23780       HEGTSESSSSDFKNIAYKFINEWRAQEPLFHSIALQQYILLCAQEQEGGLRDKPGKRRDH     392
AtERA1/At5g40280    DEDNDEDSVNGHR-IHHTSTYINRRMQLVFDSLGLQRYVLLCSKIPDGGFRDKPRKPRDF     419
                    .*...:..  ..: * :.      *  : :*.*:.**:*:***::    **:**** * **.

Glyma06g19740       YHTCYCLSGLSLCQYSWSKHPDSPPLPNLVLGPYSNLLEPIHPLFNVVLERYREAHVFFF     452
Glyma13g23780       YHTCYCLSGLSLCQYSWSKHPDSPPLPNLVLGPYSNLLEPIHPLFNVVLGRYREAHEFFF     452
AtERA1/At5g40280    YHTCYCLSGLSVAQHAWLKDEDTPPLTRDIMGGYSNLLEPVQLLHNIVMDQYNEAIEFFF     479
                    ***********:.*::* *. *:***.. ::* *******:: *.*:*: :*.**  ***

Glyma06g19740       TES     455
Glyma13g23780       TES     455
AtERA1/At5g40280    KAA     482
                    . :
```

S2 Fig. Comparison of amino acid sequences of Glyma06g19740, Glyma13g23780, and AtERA1 (At5g40280).
