# Supplementary figures and images for: Virus-induced down-regulation of GmERA1A and GmERA1B genes enhances the stomatal response to abscisic acid and drought resistance in soybean
Source: PLoS One. 2017 Apr 18;12(4):e0175650. doi: 10.1371/journal.pone.0175650 (PMC5395220; doi:10.1371/journal.pone.0175650)

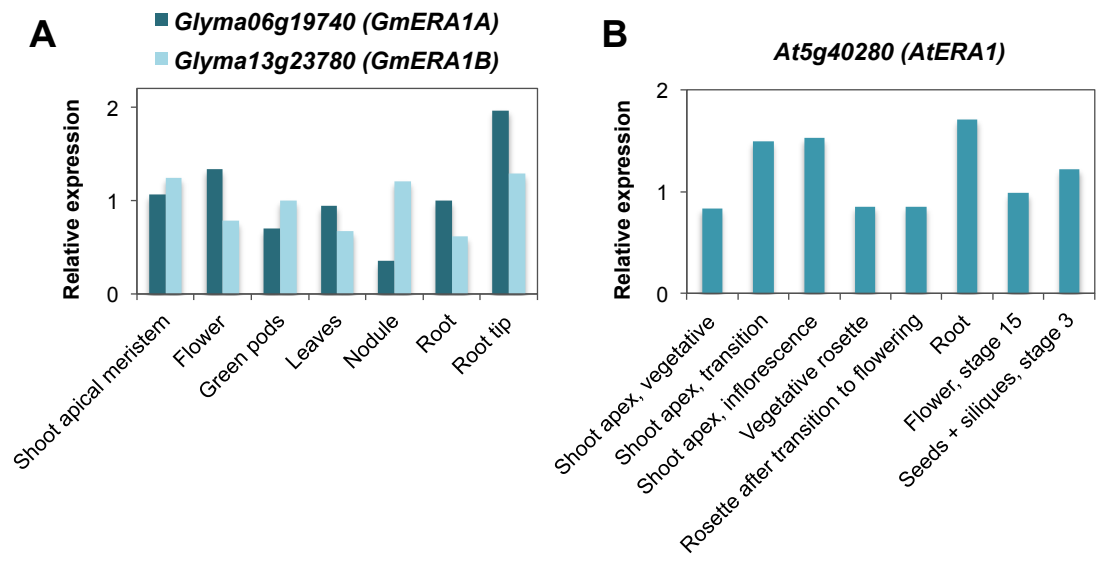

**S3 Fig. Expression status of *GmERA1A* and *GmERA1B* in soybean plants.**

Supplement: S3 Fig — mRNA expression profiles were obtained from the (A) Soybean eFP Browser and (B) Arabidopsis eFP Browser (http://bar.utoronto.ca/welcome.htm). (A) Data for the ‘Relative’ expression level of GmERA1A (Glyma06g19740) and GmERA1B (Glyma13g23780) were obtained. (B) Data for the ‘Relative’ expression level of AtERA1 (At5g40280) were obtained from the data source of ‘Developmental Map’. (PDF) [file pone.0175650.s003.pdf]

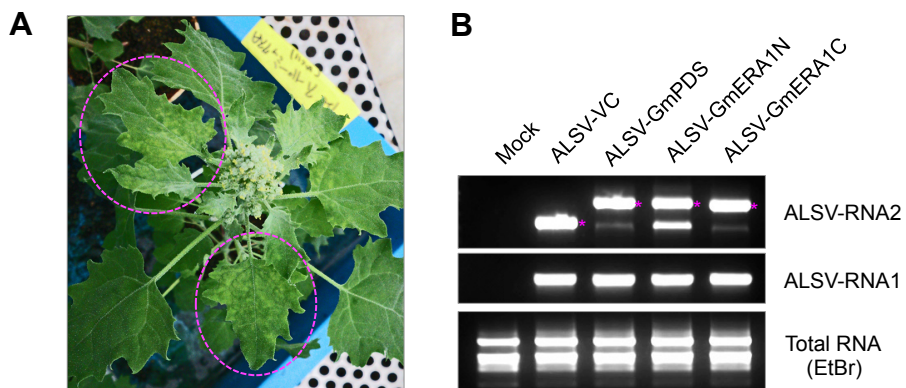

**S4 Fig. Inoculation of *Chenopodium quinoa* plants with recombinant ALSV plasmids.**

Supplement: S4 Fig — (A) Mosaic symptoms, indicating virus infection, appeared in the upper leaves of C. quinoa inoculated with ALSV plasmids approximately one week after inoculation. The photograph was taken 3 weeks after inoculation. C. quinoa leaves with mosaic symptoms (encircled with magenta dashed lines) were used for inoculum preparation. The inoculum was used for secondary inoculation of another C. quinoa plant. (B) Detection of RNA1 and RNA2 for ALSV by RT-PCR. The cDNA samples were prepared from C. quinoa leaves exhibiting mosaic symptoms. Equal amounts of total RNA (used for cDNA synthesis) were loaded as a control. Bands with asterisks (*) in ALSV-RNA2 indicate the position of the original size of each virus construct. (PDF) [file pone.0175650.s004.pdf]

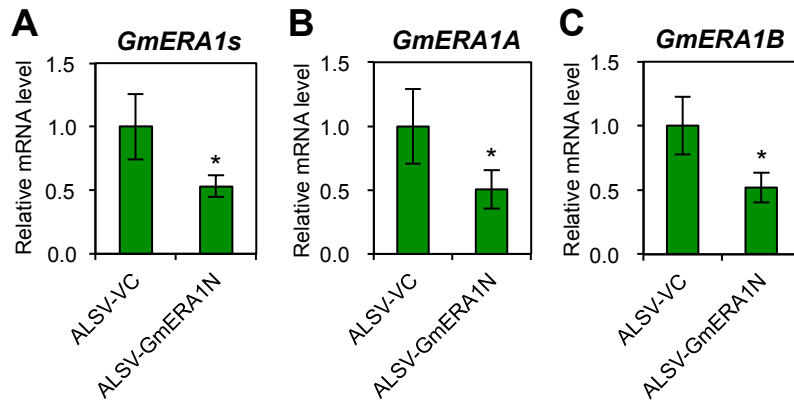

**S6 Fig. Expression analysis of *GmERA1A* and *GmERA1B* in ALSV-infected leaves.**

Supplement: S6 Fig — Total RNA samples were prepared from an attached or a 4.5 h-detached leaflet in ALSV-VC or ALSV-GmERA1N-infected soybean. (A) Both GmERA1A and GmERA1B were amplified with the same primer set (RT-F1 and RT-R1). (B) GmERA1A and (C) GmERA1B were amplified with gene-specific primer sets (RT-F2 and RT-R2 for GmERA1A; and RT-F3 and RT-R3 for GmERA1B). Relative values are presented as mean ± SD (n = 4) normalized to the expression of GmACT11 as a control. Asterisks (*) denote a significant difference from the ALSV-VC (empty vector) control by t-test (P < 0.05). (PDF) [file pone.0175650.s006.pdf]

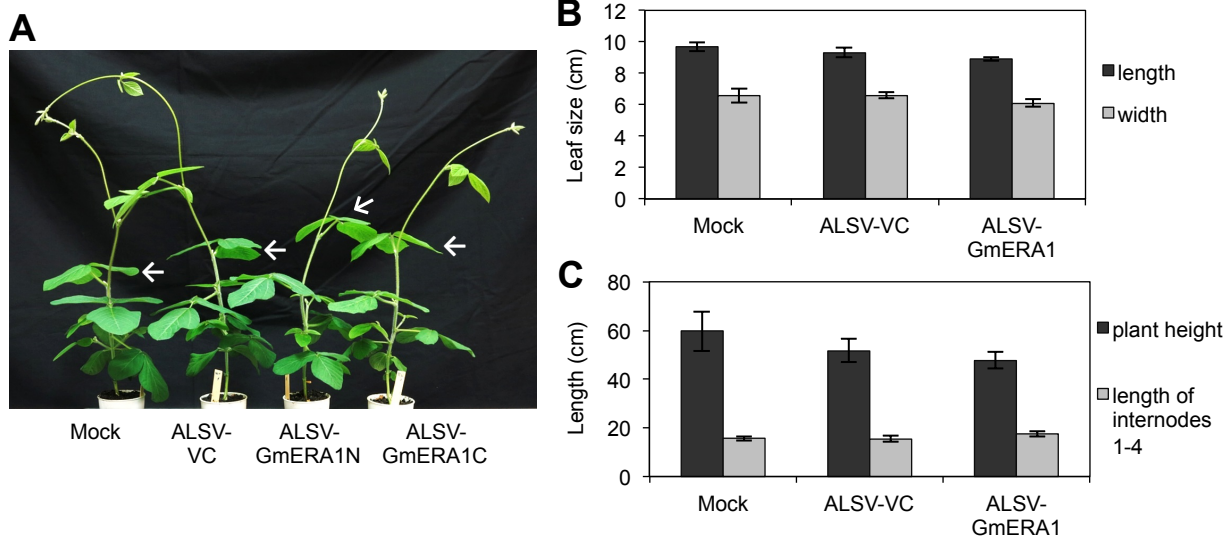

**S7 Fig. Comparison of plant sizes among soybean plants infected with ALSVs.**

Supplement: S7 Fig — (A) Plants photographed 5 weeks after inoculation with ALSV. Arrows indicate the fourth trifoliate leaves in each plant. (B and C) The size of the fourth trifoliate leaf (B) and the plant height and length of the stem from the first to the fourth internode (C) were scored 5 weeks after inoculation with ALSV (n = 4, mean ± SE). No significant difference in plant size was detected between plants inoculated with ALSV-VC and ASLV-GmERA1 at growth stages V4–V6. (PDF) [file pone.0175650.s007.pdf]

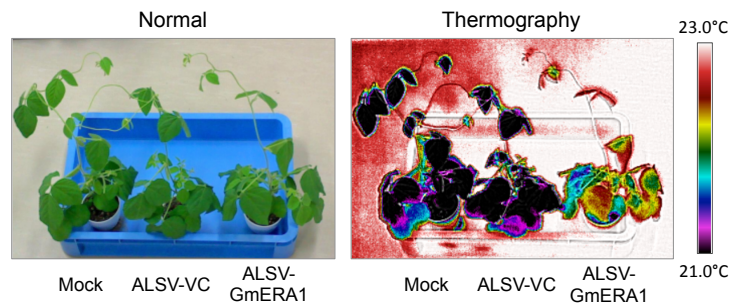

**S8 Fig. Changes in the surface temperatures of whole plants during the water withholding test.**

Supplement: S8 Fig — Watering was withheld from potted five-week-old plants at the V6 growth stage. The surface temperature of whole plants was measured at 24 h after water withholding. A representative photograph is shown. (PDF) [file pone.0175650.s008.pdf]

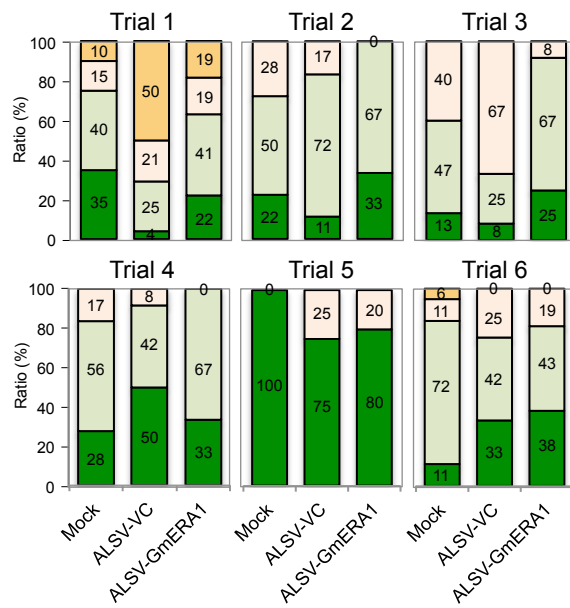

**S9 Fig. Leaf rolling scores for ALSV-infected soybean plants subjected to water withholding.**

Supplement: S9 Fig — During the water-withholding test, the drought status of the fourth to sixth trifoliate leaves was scored as described in Fig 4. Leaf drought scores at 55–62 h after water withholding are shown for six independent trials. (PDF) [file pone.0175650.s009.pdf]
