# Supplementary material for: Virus-induced down-regulation of GmERA1A and GmERA1B genes enhances the stomatal response to abscisic acid and drought resistance in soybean
Source: PLoS One. 2017 Apr 18;12(4):e0175650. doi: 10.1371/journal.pone.0175650 (PMC5395220; doi:10.1371/journal.pone.0175650)
Supplement: S5 Fig — (A) Mosaic symptoms in young soybean plants infected with ALSV. Mosaic symptoms were observed in unifoliate (left photograph) and early first-to-second trifoliate (right photograph) leaves. Representative mock-inoculated and ALSV-VC (vector control)-inoculated plants are shown. Leaves with mosaic symptoms are encircled with dashed lines. (B) Lack of mosaic symptoms and complete expansion of ALSV infection in fourth trifoliate leaves. Appreciable mosaic symptoms appeared in the third trifoliate leaves, although almost no symptoms were present in the fourth trifoliate leaves (left photograph; ALSV-VC). Bleaching symptoms caused by VIGS in soybean plants infected with ALSV-GmPDS appear to be complete in the fourth trifoliate leaves (right photograph; ALSV-GmPDS). Magenta and white dashed lines indicate fourth and third trifoliate leaves, respectively. (PDF) [file pone.0175650.s005.pdf]

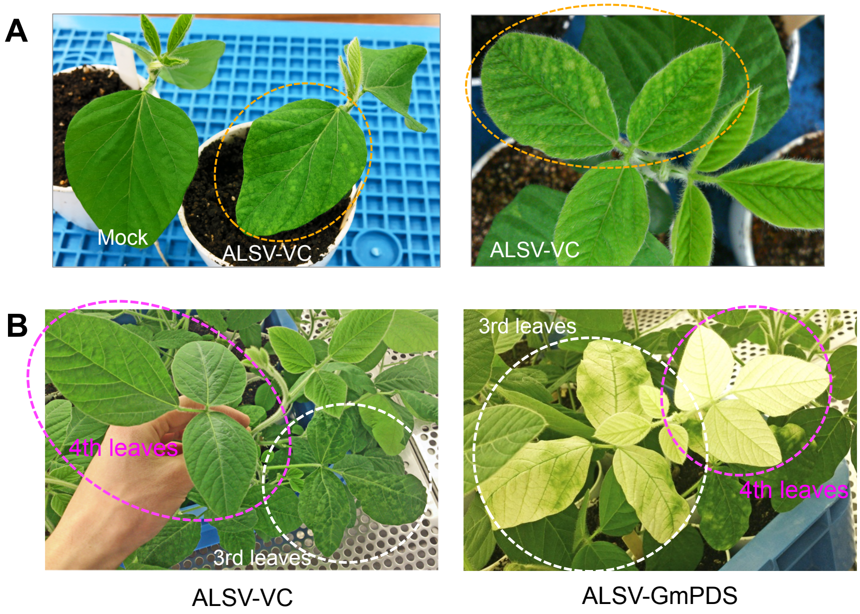

S5 Fig. Inoculation of soybean plants with recombinant ALSVs and induction of virus-induced gene silencing.
